# Identification of a Novel Mutation in the *COL2A1* Gene in a Chinese Family with Spondyloepiphyseal Dysplasia Congenita

**DOI:** 10.1371/journal.pone.0127529

**Published:** 2015-06-01

**Authors:** Xiangjun Huang, Xiong Deng, Hongbo Xu, Song Wu, Lamei Yuan, Zhijian Yang, Yan Yang, Hao Deng

**Affiliations:** 1 Center for Experimental Medicine, the Third Xiangya Hospital, Central South University, Changsha, China; 2 Department of Orthopedics, the Third Xiangya Hospital, Central South University, Changsha, China; The University of Hong Kong, HONG KONG

## Abstract

Spondyloepiphyseal dysplasia congenita (SEDC) is an autosomal dominant chondrodysplasia characterized by disproportionate short-trunk dwarfism, skeletal and vertebral deformities. Exome sequencing and Sanger sequencing were performed in a Chinese Han family with typical SEDC, and a novel mutation, c.620G>A (p.Gly207Glu), in the collagen type II alpha-1 gene (*COL2A1*) was identified. The mutation may impair protein stability, and lead to dysfunction of type II collagen. Family-based study suggested that the mutation is a *de novo* mutation. Our study extends the mutation spectrum of SEDC and confirms genotype-phenotype relationship between mutations at glycine in the triple helix of the alpha-1(II) chains of the *COL2A1* and clinical findings of SEDC, which may be helpful in the genetic counseling of patients with SEDC.

## Introduction

Spondyloepiphyseal dysplasia congenita (SEDC, OMIM 183900) is a common subtype of the type II collagenopathies, a heterogeneous group of chondrodysplasias resulting from mutations in the collagen type II alpha-1 gene (*COL2A1*, OMIM 120140) [1]. The disorder was first described in 1966 with a low prevalence of 3.4/1,000,000 [2,3]. It is characterized by disproportionate short-trunk dwarfism, skeletal and vertebral deformities, including scoliosis and thoracic hyperkyphosis, coxa vara, avascular necrosis-like changes in bilateral femoral epiphyses, hip pain or waddling gait, genu valgum, and various joint diseases [3–5]. Skeletal deformities are the most commonly shared features in SEDC. Extraskeletal manifestations, including hearing loss, cleft palate and ocular anomalies, have also been reported in some cases [3,6]. To date, mutations in the *COL2A1* gene have been reported to be responsible for at least 16 subtypes of type II collagenopathies, including achondrogenesis, type II or hypochondrogenesis (ACG2/HCG, OMIM 200610), platyspondylic skeletal dysplasia, Torrance type (PLSDT, OMIM 151210), spondyloperipheral dysplasia (SPD, OMIM 271700), SEDC, spondyloepimetaphyseal dysplasia, Strudwick type (SEMDSTWK, OMIM 184250), Kniest dysplasia (OMIM 156550), Stickler syndrome, type I (STL1, OMIM 108300), Stickler syndrome, type I, nonsyndromic ocular (OMIM 609508), osteoarthritis with mild chondrodysplasia (OMIM 604864), avascular necrosis of the femoral head (ANFH, OMIM 608805), Legg-Calve-Perthes disease (LCPD, OMIM 150600), epiphyseal dysplasia, multiple with myopia and deafness (EDMMD, OMIM 132450), otospondylomegaepiphyseal dysplasia (OSMED, OMIM 215150), Czech dysplasia (OMIM 609162), spondyloepiphyseal dysplasia, Namaqualand type (SEDN), and vitreoretinopathy with phalangeal epiphyseal dysplasia (VPED) [7–9].

The aim of this study was to identify causal gene responsible for familial SEDC in a four-generation Chinese pedigree. A novel *COL2A1* gene mutation, c.620G>A (p.Gly207Glu), was identified, resulting in a change of conserved glycine in the triple helix of the alpha-1(II) chains. Substitution of the highly conserved amino acid may impair protein stability and lead to dysfunction of helical structure and type II collagen [10].

## Materials and Methods

### Participators and clinical evaluation

**Fig 1:**
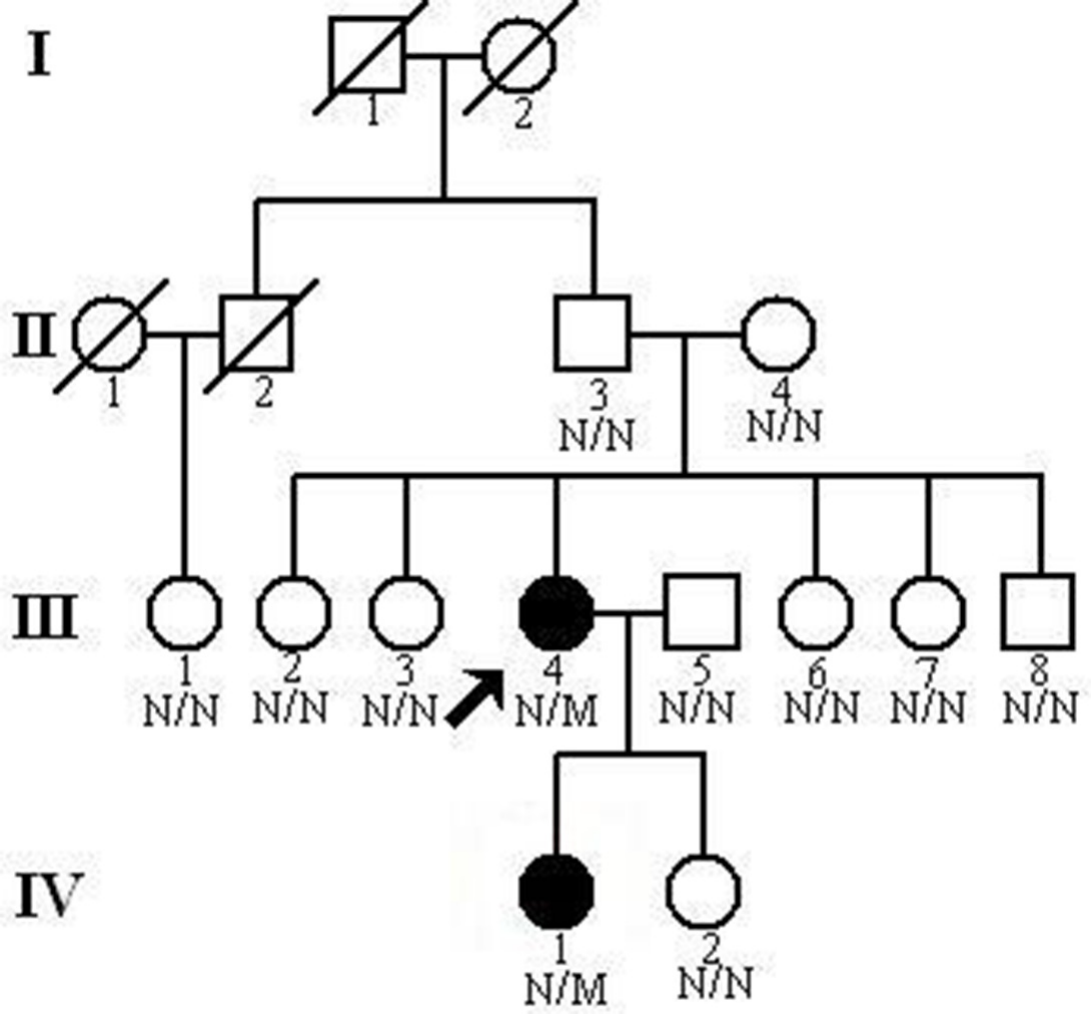
Pedigree of the family with spondyloepiphyseal dysplasia congenita showing affected cases (fully shaded). N: normal; M: the *COL2A1* c.620G>A (p.Gly207Glu) mutation.

The participating individuals or their guardians provided written informed consent for this study and the approval for the research was received from the Ethics Committee of the Third Xiangya Hospital, Central South University, China. A four-generation, 16-member Chinese Han family with familial SEDC was recruited from the Third Xiangya Hospital, Central South University, China (Fig 1). Peripheral blood samples were taken from 12 family members, and detailed clinical records of medical history, physical examinations and laboratory analyses were obtained from available family members. Blood samples were also collected from 100 unrelated ethnically-matched healthy control volunteers with negative family history of this disorder (male/female: 50/50, age 32.2±8.3 years).

### Exome capture

Extraction of genomic DNA was performed by using standard phenol-chloroform extraction method from peripheral blood lymphocytes [11]. Three micrograms (μg) of genomic DNA was used to establish the exome library. Genomic DNA of the proband (III:4) was randomly sheared by sonication and then hybridized to the Nimblegen SeqCap EZ Library to enrich exonic DNA in each library, following the manufacturer’s instructions. Enriched library targeting the exome was sequenced on the Illumina HiSeq 2000 platform to generate 90-bp paired-end reads [12]. A mean exome coverage of 77.52× was obtained, allowing for the exam of selected region and sufficient depth to accurately match 99.46% of targeted exome [11].

### Read mapping and variant analysis

The available human reference genomic sequences were accessed from the public online UCSC database (http://genome.ucsc.edu/), version hg19 (build 37.1). Sequences obtained from the proband were aligned using SOAPaligner (soap2.21), and single nucleotide polymorphisms (SNPs) were sifted out using SOAPsnp with the defined parameters after the deletion of duplicated reads (obtained mainly in the PCR step). Small insertions or deletions (indels) in the coding sequence and splicing sites were prosecuted [12–14]. Candidate SNPs were filtered by the following criteria: SNP quality ≥20 (score 20 represents 99% accuracy of a base call), sequencing depth ≥4, the estimated copy number ≤2, and the distance between two SNPs >5 [15]. The common variants and non-pathogenic candidate variants were filtered against several public databases including the Single Nucleotide Polymorphism database (dbSNP build 137, http://www.ncbi.nlm.nih.gov/projects/SNP/snp_summary.cgi), 1000 genomes project (1000genomes release_20100804, http://www.1000genomes.org/), HapMap project (2010–08_phase II + III, http://hapmap.ncbi.nlm.nih.gov/), and YanHuang (http://yh.genomics.org.cn/) project.

Multiple alignment of the protein sequence was performed across different species by the Basic Local Alignment Search Tool (http://blast.st-va.ncbi.nlm.nih.gov/Blast.cgi). Online tools including Polymorphism Phenotyping version 2 (PolyPhen-2, http://genetics.bwh.harvard.edu/pph2/), Sorting Intolerant From Tolerant (SIFT, http://sift.jcvi.org/, score less than 0.05 is deleterious) and MutationTaster (http://www.mutationtaster.org/) were used to evaluate whether amino acid substitutions affect protein function [16–18]. Locus-specific primers used for PCR amplification and direct sequencing were designed using the online Primer3 program (http://primer3.ut.ee/). Then Sanger sequencing of PCR products was performed to validate the potential disease-causing variants with ABI3500 sequencer (Applied Biosystems Inc., Foster City, CA, USA), according to the procedures described previously [11]. Primer sequences used for the *COL2A1* gene causative variant were as follows: 5′-GCTTGGGAATCATCTGCGAC-3′ and 5′-TGGGAAATGAGAGGGAGCAG-3′.

## Results

### Clinical findings

Affected individuals (III:4 and IV:1) showed similar clinical and radiological abnormalities. Patients had short-trunk dwarfism, short neck, spinal and limbs deformities, coxa vara, genu valgum, waddling gait and various joint diseases, especially loss of complete bone formation of femoral head. Short-trunk dwarfism was noticed at birth in these two patients, and the growth was found to be markedly delayed. While cleft palate, ocular complications, hearing loss and other tube malformation were absent in both patients. Skeletal radiographs showed odontoid hypoplasia, atlantoaxial subluxation, scoliosis, kyphosis, lumbar lordosis and ovoid vertebral bodies, capital femoral epiphyses dysplasia, flattening of the acetabular roof, shortening of the femoral neck and base of the ilium, and delayed fusion of the pubic and ischial bones. No SEDC-related clinical or radiological abnormalities were observed in other recruited family members.

### Mutation screening

We performed exome sequencing on genomic DNA sample of the proband (III:4) of the Chinese Han family with SEDC. A total of 6.80 billion bases of sequence with a 90-bp read length from the patient were generated, and 6.62 billion bases (97.38%) passed the quality assessment. Among them, 6.19 billion bases (93.50%) were aligned to the human reference sequence and 4.33 billion bases covered the target region with a mean coverage of 77.52-fold [12]. 110,479 genetic variants, including 14,897 non-synonymous changes, were occurred at the coding sequence or the canonical dinucleotide of the splice site junctions. A filtration strategy was performed to detect the responsible mutation for the disorder, following the priorization scheme used in recent studies [11]. Given that the disorder frequency is <0.005, we excluded known variants recorded in dbSNP137, 1000 genomes project, HapMap or Yanhuang projects with MAF >0.50%. Using the above filtering strategy, the number of candidate genes was reduced by more than 93.35%. Sequence variants were also filtered by in-house database from BGI-Shenzhen. Subsequently, a missense mutation in the *COL2A1* gene, the potential disease-causing gene of SEDC, was selected for further validation.

**Fig 2:**
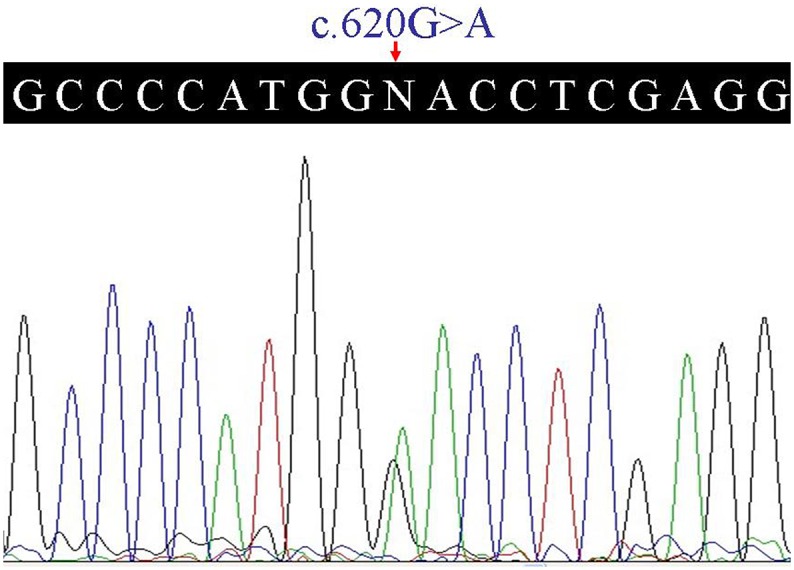
Heterozygous c.620G>A (p.Gly207Glu) mutation in the *COL2A1* gene.

**Fig 3:**
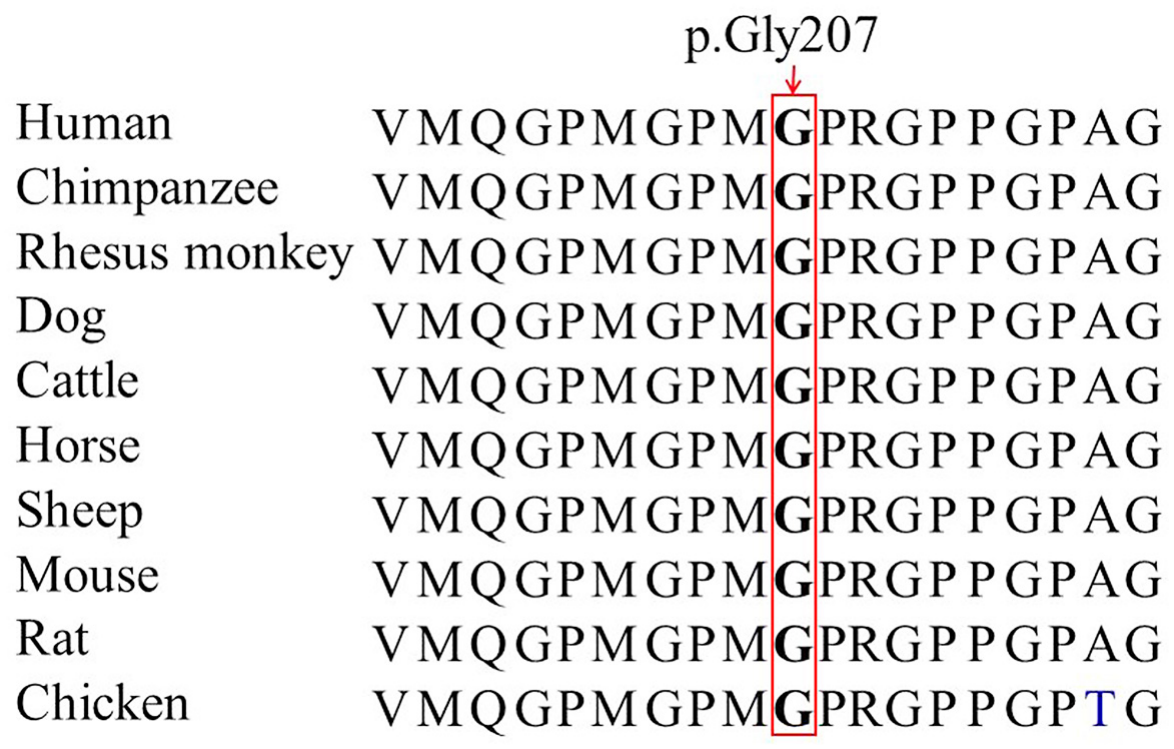
Conservation analysis of the COL2A1 p.Gly207 amino acid residue.

After validation by Sanger sequencing and comparison with mutation from the Human Gene Mutation Database (http://www.hgmd.cf.ac.uk/), a novel variant, c.620G>A (p.Gly207Glu), in the *COL2A1* gene was observed in the proband. The c.620G>A variant was subsequently identified in her affected daughter (IV:1) (Fig 2), and validated for the absence in unaffected family individuals and 100 unrelated normal controls. The co-segregation of variant with this disease and the bioinformation analysis suggest that this variant is likely the pathogenic mutation. The glycine at position 207 is phylogenetically conserved among various species based on multiple sequence alignment (Fig 3). PolyPhen-2 analysis predicted to be probably damaging with a score of 1.00 on HumVar database (sensitivity: 0.00; specificity: 1.00). The SIFT prediction also showed a damaging effect with a score of 0.00. MutationTaster predicted that the alteration was disease-causing with a probability value close to 1 indicating the high security of prediction. Computer-based protein analysis indicates that the variant in the *COL2A1* gene was likely deleterious and the disease-causing mutation in our family.

## Discussion

SEDC, a rare but differentiable skeletal malformation caused by mutations in the *COL2A1* gene, is usually evidenced at birth [19]. It is characterized by short-trunk dwarfism, abnormal epiphyses, and platyspondyly [20,21]. SEDC, including both mild and severe coxa vara (SEDC-M and SEDC-S), is the most severe form of hypogenesis of the femoral head. SEDC can be distinguished from other diseases by its characteristic manifestations, including platyspondyly, odontoid hypoplasia, normal hands and feet, and extraskeletal features, including myopia, sensor neural hearing loss, bifid uvula, cleft palate and hypospadias [6,7]. It is pathologically characterized by PAS-positive cytoplasmic inclusions and cystic spaces in resting cartilage [22]. Though SEDC is an autosomal dominant skeletal disorder, most cases of SEDC are sporadic [6]. Clinical variability exists in the SEDC, even the same point mutation can cause different manifestations [20].

In this study, a heterozygous missense mutation c.620G>A (p.Gly207Glu), in the *COL2A1* gene was identified in a Chinese Han family with SEDC by exome sequencing and Sanger sequencing. Two patients (III:4 and IV:1, Fig 1) showed typical clinical features of SEDC-M, including short-trunk dwarfism, spinal and limbs deformities. Neither patient showed any abnormalities in the eye, auditory system or other tube bones. The mutation co-segregated with the disorder in the family and was absent in unaffected family members, 100 normal controls, and public databases. Multiple sequence alignment showed that the mutated glycine at position 207 is phylogenetically conserved, and computer-based analysis revealed that the mutation is likely deleterious, supporting that the p.Gly207Glu variant is likely pathogenic. Given that both parents (II:3 and II:4, Fig 1) of the proband were free of p.Gly207Glu mutation and had no clinical evidence for SEDC, the mutation was thought to be a *de novo* mutation.

The *COL2A1* gene, located on chromosome 12q13, consists of 54 exons spanning over 31.5 kb of human genomic DNA and encodes a 134.4 kDa protein with 1,487 amino acids. The collagen triple helical domain characterized by three identical polypeptide chains (Gly-X-Y) is the backbone of the COL2A1 molecule, and the alpha-chain helical region is composed of 1,014 amino acids with glycine occupying every third positions, critical to correct folding and stability of the helix [23,24]. The protein is synthesized as large procollagen chains containing N- and C-terminal amino acid sequences named propeptides [25], and the C-terminal non-collagenous domain (designated as C-propeptide), which is necessary for chain association and subsequent triple helix formation [26]. After secreted into the extracellular matrix, the propeptides are cleaved and form the mature type II collagen [25]. Type II collagen co-aggregates with collagen XI, which is covalently linked to collagen IX and interacts with small leucine-rich proteoglycans influencing collagen fibrillar structure and function [23]. The p.Gly207Glu mutation led to change of a neutral amino acid residue, glycine, to a negatively charged residue, glutamic acid, involving in the basic repeating unit of the triple helical domain, the Gly position of a Gly-X-Y triplet. The substitution may impair protein stability and thus helical structure and proper function of the protein, leading to the disorder [10,27,28].

Up to now, including the p.Gly207Glu mutation, at least 35 different mutations in the *COL2A1* gene have been described to be related to SEDC with a broad phenotypic spectrum in various ethnic groups. These mutations include missense, deletion, duplication and splicing mutations, and the p.R989C mutation appears to be a hot spot mutation with a strikingly similar phenotype [1,5,29]. The accurate relationship between the *COL2A1* gene mutations and corresponding phenotypes is far from clear. The expression profiles and characteristics of mutations can also be determined by genetic background [21].

The *COL2A1* mutations most likely produce structurally abnormal type II collagen in the extracellular matrix, hampering endochondral ossification and linear bone growth by a dominant negative mechanism [30]. The glycine mutation in the triple-helical domain may harm the intracellular transport and collagen secretion, and glycine-to-nonserine residue substitutions have been reported to create more severe phenotypes, consistent with the observation in our patients [7,31].

## Conclusions

A novel *de novo* p.Gly207Glu mutation was identified in our family with SEDC, which was suggested to be the genetic cause and extends the mutation spectrum of the *COL2A1* gene. Whole exome sequencing provides a cost-effective and expedited approach to identify pathogenic mutations responsible for inherited disorders. Our finding may provide new insights and approaches into the genetic cause and diagnosis of SEDC, and may also have implications for genetic counseling and clinical intervention. More extensive studies of the *COL2A1* including in larger and diverse ethnic groups are warranted to explore the underlying pathogenic mechanism of SEDC and elucidate the potential genotype-phenotype relationship, which in turn may supplement our understanding of type II collagenopathies [3,6].
